# Henipavirus evidence gaps: a Rapid Research Needs Appraisal

**DOI:** 10.1136/bmjph-2025-004195

**Published:** 2026-07-06

**Authors:** Marieke de Swart, Amen-Patrick Nwosu, Sandrena Ruth Frischer, Tara Hurst, Yonela Ntamo, Dijana Spasenoska, Md Zakiul Hassan, Marakiya Moetlediwa, Daisy Mdumei Mpando, Brian Buckley, Gemma Villanueva, Nicholas Henschke, Katrin Probyn, Eli Harriss, Sophia Adhikari, Vincent Cheng, Duduzile Ndwandwe, Alice Norton, Louise Sigfrid

**Affiliations:** 1 Policy and Practice Research Group, Pandemic Sciences Institute, University of Oxford, Oxford, UK; 2 Pandemic Sciences Institute, University of Oxford, Oxford, UK; 3 Cochrane South Africa, Tygerberg, South Africa; 4 Programme for Emerging Infections, Infectious Diseases Division, International Centre for Diarrhoeal Disease Research Bangladesh, Dhaka, Bangladesh; 5 Cochrane Response, Cochrane, London, UK; 6 Bodleian Health Care Libraries, University of Oxford, Oxford, UK; 7 NHS Foundation Trust, Bristol, UK; 8 South African Medical Research Council, Cape Town, South Africa

**Keywords:** Public Health, Disease Outbreaks, Systematic Review

## Abstract

**Introduction:**

Henipaviruses have been identified as priority pathogens by the WHO for research and development. We applied a rapid research needs appraisal (RRNA) review methodology to systematically identify existing henipavirus evidence on clinical characteristics, immune response, transmission, risk factors, medical countermeasures and associated social and behavioural factors, to inform research prioritisation, and strategies to strengthen our preparedness and response for henipaviruses to protect populations and improve outcomes.

**Method:**

We searched PubMed, Ovid Embase, Cochrane Library, Epistemonikos from 1974 to 15 May 2025 for studies focused on henipavirus infection including humans or human samples. Two reviewers screened studies and extracted data across predefined research domains without language restrictions. Data were analysed descriptively.

**Results:**

Of 106 records included, most focused on Nipah (82.1%, 87/106), followed by Hendra (17.9%, 19/106) and Langya virus (1.9%, 2/106) disease, set in Asia (82.1%, 87/106), Australia (17.9%, 19/106) and Europe (0.9%, 1/106). Most (96.2%, 102/106) were observational, 3.8% (4/106) interventional studies. Adults were included in 76.4% (81/106) studies, children in 32.1% (34/106); pregnant women in 0.9% (1/106). Studies focused on clinical characteristics (n=59), transmission (n=28), risk factors (n=16), social science (n=17), diagnostics (n=14), immune response (n=9), and therapeutics (n=4). Of the four interventional studies, two focused on medical countermeasures, including a non-randomised controlled Nipah ribavirin study and a phase I m102.4 antibody Nipah and Hendra treatment trial in adults. Two were social science studies: one a self-instruction prevention training module, the other a workshop on personal protective equipment. Human vaccine studies were lacking, but nine studies explored attitudes to vaccination of horses against Hendra virus.

**Conclusion:**

Our data highlight the limited evidence available and the lack of medical countermeasures for henipaviruses. The gaps in inclusion of high-risk populations (young children, pregnant women) are concerning. Our data show a need to identify effective medical countermeasures, supported by behavioural studies, and to invest in One Health studies to generate evidence to inform strategies to protect societies against the threat of henipaviruses.

**WHAT IS ALREADY KNOWN ON THIS TOPIC**

- Nipah virus has been identified as a high-priority pathogen with potential to constitute a Public Health Emergency of International Concern by the WHO, due to its high mortality and morbidity risk, and lack of available medical countermeasures.
- In the WHO pathogens prioritisation framework, another member of the *Henipavirus* genus, Hendra virus, is proposed as a model for pathogen X research and development.
- Effective research responses to high consequence outbreaks such as Nipah require prioritisation, preparation, and coordination in preparedness time to be ready to implement at the onset of an outbreak.

**WHAT THIS STUDY ADDS**

- We present an overview of existing published evidence on henipaviruses detected to cause human disease across key domains relevant to prevention, health protection, clinical and public health management, highlighting the limited evidence available to inform evidence-based clinical and public health management. Our data highlight significant gaps in our understanding of henipaviruses from community, public health and health service perspectives, including on risk factors for severe disease, transmission dynamics, immune protection from natural infection, optimal prevention, supportive care and treatment strategies, medical countermeasures.
- We also identified limited evidence on awareness of the disease, risk factors and prevention and control methods among at-risk communities. No studies on strain-specific diagnostic tests were identified.

**HOW THIS STUDY MIGHT AFFECT RESEARCH, PRACTICE OR POLICY**

- Our data present an overview of the evidence available for henipaviruses of public health significance of relevance to inform clinical, public health and research strategies.
- The risk of emergence of new henipaviruses, such as Langya virus disease, together with the limited availability of rapid diagnostics, prophylaxis, medical countermeasures and awareness is a cause for concern. The data show a need to strengthen our preparedness for implementation of studies to address the evidence gaps identified.
- Our data provide a baseline to inform research prioritisation and coordination, as well as clinical and public health strategies to protect populations and improve outcomes.

##  INTRODUCTION

The *Henipavirus* genus contains priority pathogens recognised by WHO for research and development.^1^ Henipaviruses are single-stranded, negative sense RNA viruses belonging to the Paramyxoviridae family. Nipah virus (NiV, *Henipavirus nipahense*) has caused regular outbreaks in pigs and humans in countries in Southeast Asia, with an estimated human case fatality rate (CFR) of 61%.^2^ Hendra virus (HeV, *Henipavirus hendraense*) has caused outbreaks predominantly in Australia since 1994, with an estimated human CFR of 57%.^3^ Fruit bats (genus: *Pteropus*)*,* also known as flying foxes, are NiV and HeV reservoirs.^4 5^ Several intermediate species are known to carry NiV such as pigs, dogs, cats and monkeys.^6 7^ These viruses have spilled over to humans via consumption of food or drink contaminated with bat secretions, or exposure to an infected intermediate species.^2^

Human Nipah virus infection was first detected in 1998, following an outbreak in pigs and humans in Malaysia and Singapore.^8^ There were 265 human cases with encephalitis with a CFR of 40% (105/265) reported.^8^ More than 1 million pigs were culled to control the outbreak, resulting in substantial economic loss.^9^ Since 2001, there have been regular NiV outbreaks in Bangladesh and India, with an overall mortality of 62.1% and 82.7%, respectively.^10^ An outbreak in 2023 in Bangladesh primarily affected a young population, with a median age of 16 years (range: 15 days to 50 years).^11^

NiV has three predominant genotypes: Bangladesh (NiV-B), Malaysia (NiV-M) and India (NiV-I).^12^ Historically, the Bangladesh–India region has reported a higher CFR (70%) compared with the Malaysia–Singapore region (32–41%), which may reflect differences in strain and/or health systems, such as variation in detection, reporting, access to critical and intensive care.^13^,^15^ During an outbreak in 2023, in Kerala, India the reported CFR was 33%, which was attributed to the public health infrastructure and swift response measures.^16^ In humans, Nipah virus infection has an incubation period of 414 days. Presentation ranges from asymptomatic to severe acute respiratory illness or encephalitis.^17^ Post-acute neurological sequelae have been described in survivors, but data are limited.^18^

HeV has caused spillover Hendra virus disease in humans from horses. HeV was first isolated in 1994 in Brisbane, Australia, during an outbreak affecting 21 horses and two humans.^19^ In total, seven human cases, all adults, have been reported, with the most recent case detected in 2009.^5^ HeV infection has an incubation period of 5–21 days in humans. It generally presents with influenza-like illness, in some progressing to encephalitis, although a single case of encephalitis was reported without prior influenza-like illness.^20^ Neurological sequelae have been reported.^21^ HeV has only been detected in Australia, although fruit bats with HeV-specific antibodies have been identified in Indonesia.^22^ A new HeV variant (HeV-g2) was isolated from an infected horse in 2022, but has not been detected in humans.^22^

Langya virus (LayV) is a henipavirus identified in 35 cases in China since 2018, with shrews identified as the probable natural reservoir.^23^ It is associated with febrile illness. The sample sizes have been too small to determine the risk of human-to-human transmission.^24^

NiV is designated a high priority pathogen due to the high morbidity and mortality risk, together with a lack of medical countermeasures, unpredictable spillover events, human-to-human transmission, and a potential risk of emergence into new regions due to a wide distribution of the reservoir host.^25^ No licensed therapeutics or vaccines are available for humans. However, a vaccine against HeV is licensed for use in horses (Equivac HeV), and HeV-sG-V, a human version based on the same antigen, has been tested in primates.^26^ There are a few vaccines registered in phase I trials: two against NiV; ChAdOx1-NiVB^27^ and mRNA-1215 (Moderna/NIAID),^28^ and one for HeV, HeV-sG-V (Auro Vaccines/PATH).^29^ With a lack of medical countermeasures, prevention of zoonotic spillover, early detection, isolation of cases and infection prevention control and optimal supportive care are key for reducing risk of transmission and improving survival rates.

Harmonisation of research efforts for priority pathogens is required, as evidenced by the many trials that failed to reach significant results during the COVID-19 pandemic^30 31^ due to a lack of coordination and underpowered study designs. Achieving this requires planning and research prioritisation, as well as collaboration, to inform targeted, well-designed research protocols, pre-positioned to be implemented as a new outbreak emerges, to identify evidence with capacity to inform prevention, management and control strategies. Implementing research for new and (re-)emerging infectious disease is challenging, as emergence and outbreak trajectories are hard to predict.^32^ It is key to ensure that resources are prioritised to ensure research is focused on addressing key needs for the whole population in high-risk areas. Towards this aim, we established a rapid research needs appraisal (RRNA) platform for priority infectious diseases of public health significance.^33^ We developed the RRNA method to strengthen capacity to robustly and rapidly appraise all existing published evidence across a set of key predefined research domains. Our aim is to identify existing evidence and gaps in the henipavirus evidence base across research categories, population groups and settings to inform management and coordination of research strategies to prevent transmission and improve disease outcomes.

## Methods

### Study design

We used a rapid RRNA^33^ to identify existing evidence across predefined research domains targeting our generic RRNA protocol to henipaviruses (osf/s8mt5).^34^ The research domain categories were identified by a multidisciplinary panel as key for informing prevention, early protection of communities, frontline health workers, clinical management and outbreak control.^33^ The manuscript is reported following Preferred Reporting Items for Systematic Reviews and Meta-Analyses (PRISMA).^35^

### Search strategy and selection criteria

We searched four databases, MEDLINE (PubMed), Embase (OVID), Cochrane Library, Epistemonikos and clinicaltrials.gov from 1974 to 15 May 2025. The main search terms were “Henipavirus” OR “Henipavirus Infections” OR nipah* OR hendra OR NiV (online supplemental appendix 1: search strategy). We included studies that reported primary evidence on humans or human samples and at least one of our nine research domain categories: clinical characteristics, transmission, risk factors, diagnostics, prophylaxis, immune protection, therapeutics, supportive care and associated social, behavioural factors. We excluded narrative reviews, book chapters, pre-prints, conference abstracts and opinion pieces (not presenting primary data), ongoing trials, and pure animal and cell culture studies. There were no exclusions by language.

### Study selection and data extraction

Screening and data extraction was performed using previously piloted screening and data extraction forms in the systematic review (SR) software DistillerSR.^36^ Two reviewers independently screened titles and abstracts for both inclusion and exclusion; disagreements were resolved via consensus or a third reviewer. Full-text screening and data extraction were combined, using an accelerated screening approach, whereby one reviewer screened articles for inclusion and two for exclusion. If a study was included, the data were extracted at this stage, to reduce risk of over-inclusion. Full-text articles not accessible via Endnote’s automatic retrieval functions,^37^ or manually via university access, PubMED or Google were excluded as inaccessible.

We extracted data on bibliography, study design, setting, population and the research domain questions (online supplemental appendix 2). The RRNA includes a data prioritisation step, whereby studies of lower level of study design, such as case reports, are included, but not extracted if there is higher level of evidence available addressing the research domain question. Instead, these are listed with their bibliography in a supplemental file (online supplemental appendix 3). We defined cases as laboratory or clinically confirmed and probable cases or serologically confirmed past infection as reported by the study authors. Probable cases are defined as those cases where the diagnosis is the most likely one based on clinical symptoms, symptom onset, case contact and ongoing outbreaks, in the absence of laboratory confirmed diagnosis. Age groups were defined as neonates (≤28 days), infants (29 days to 12 months), young children (>1–5 years), older children (6–17 years), adults (18–64 years) and elderly (≥65 years).

### Data analysis

Data were analysed descriptively. We did not perform a meta-analysis nor a formal risk of bias analysis, as this is out of scope for the RRNA methodology, where the objective is to enable rapid, systematic identification of evidence for gaps analysis across a wide range of study design. Instead, included studies were critically appraised in the narrative by study design, number of cases included, and reporting of demographics data. Figures were created using Microsoft Office and RStudio V.4.3.2 ggplot2 packages.^38^

## Results

**Figure 1:**
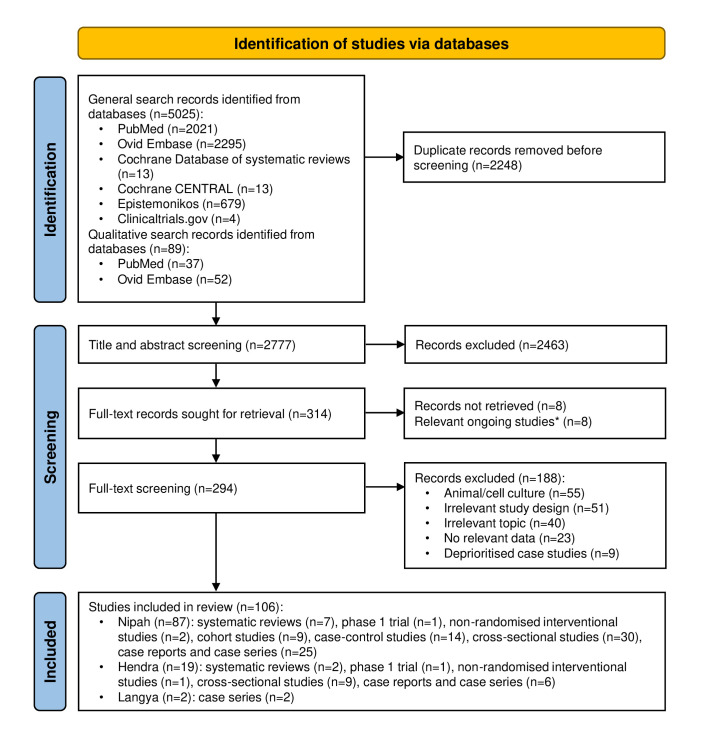
Preferred Reporting Items for Systematic Reviews and Meta-Analyses (PRISMA) flow diagram. *Identified, but deprioritised from data extraction due to higher level of evidence available for the domain.

Of the 3987 records screened, 106 studies were eligible and included (figure 1, online supplemental appendix 4). Of these, 6.6% (7/106) were SRs, 2.8% (3/106) non-randomised interventional studies, 0.9% (1/106) phase I trial, 8.5% (9/106) cohort studies, 13.2% (14/106) case–control studies, 37.7% (40/106) cross-sectional studies and 31.3% (32/106) case series and reports (table 1). Nine case series and reports with less than 10 participants were deprioritised due to higher levels of evidence being available (online supplemental appendix 3).

**Table 1:** Overview of the included studies by domain and disease

Domain (n)*	Study design (n)	Setting (n)*	Inclusions†	Age groups (n)‡	Research topics (n)*
*NiV (n=87)*					
Clinical characteristics (n=53)	Systematic reviews (n=4) Cohort (n=6) Case–control (n=6) Cross-sectional (n=18) Case series/reports (n=19)	Australia (n=1); Bangladesh (n=23); India (n=18); Malaysia (n=12); Philippines (n=2); Singapore (n=9)	Total: 8078 Cases: 4739	Young children (n=5) Older children (n=24) Adults (n=41) Elderly (n=9)	Strains (n=4); signs and symptoms (n=27); laboratory features (n=10); clinical differential features (n=1); clinical features to assess severity (n=3); asymptomatic infection (n=8); case fatality ratio (n=35); long-term complications (n=13)
Transmission and prevention (n=25)	Systematic reviews (n=3) Cohort (n=2) Case–control (n=7) Cross-sectional (n=8) Case series/reports (n=5)	Australia (n=1); Bangladesh (n=14); Germany (n=1); India (n=9); Japan (n=1); Malaysia (n=5); Philippines (n=1); Singapore (n=4); NR (n=2)	Total: 2560 Cases: 1977	Young children (n=4) Older children (n=10) Adults (n=18) Elderly (n=3)	Incubation period (n=9); transmission routes (n=16); infective body fluids (n=35); virus inactivation methods (n=2)
Risk factors (n=16)	Systematic review (n=1) Cohort (n=2) Case–control (n=6) Cross-sectional (n=5) Case series/reports (n=2)	Bangladesh (n=10); India (n=1); Malaysia (n=6); Philippines (n=2); Singapore (n=1)	Total: 4347 Cases: 3159	Young children (n=5) Older children (n=10) Adults (n=14)	Risk factors for infection (n=12); risk factors for severe disease (n=3); risk factors for mortality (n=1)
Diagnostics (n=14)	Case–control (n=3) Cross-sectional (n=6) Case series/reports (n=5)	Bangladesh (n=6); Cambodia (n=1); Germany (n=1); India (n=5); Singapore (n=1)	Total: 2852 Cases: 714	Young children (n=2) Older children (n=6) Adults (n=8) Elderly (n=2)	Diagnostic test types (n=14); sensitivity and specificity (n=3); body fluids tested (n=14)
Immune protection (n=9)	Cohort (n=1) Case–control (n=1) Cross-sectional (n=4) Case series/reports (n=3)	Bangladesh (n=2); India (n=3); Indonesia (n=1); Malaysia (n=2); Singapore (n=1)	Total: 398 Cases: 157	Neonates (n=1) Young children (n=1) Older children (n=4) Adults (n=8)	Vertical transfer of human immunity (n=1); seroprevalence (n=IgG, IgM) (n=3); seroprevalence without follow-up (n=2); patients vaccinated against JEV (n=1)
Therapeutics (n=4)	Phase I trial (n=1) Interventional (n=1) Cross-sectional (n=1) Case series/reports (n=1)	Australia (n=1); Malaysia (n=2); Singapore (n=1)	Total: 339 Cases: 245	Older children (n=1) Adults (n=4) Elderly (n=1)	RCT for m102.4 antibodies (n=1); ribavirin effectiveness (n=2); acyclovir administration (n=1)
Supportive care (n=3)	Cohort (n=1) Cross-sectional (n=1) Case series/reports (n=1)	Malaysia (n=3)	Total: 200 Cases: 200	Young children (n=1) Older children (n=2) Adults (n=3) Elderly (n=2)	Use of ventilation (n=3); seizure treatment (IV phenytoin) (n=1); vasculitis-induced thrombosis treatment (n=1)
Social sciences (n=6)	Interventional (n=1) Cross-sectional (n=5)	Bangladesh (n=4); India (n=2)	Total: 4921 Cases: 0	Adults (n=6)	Disease knowledge (n=5); identifying or managing disease (n=3)
*HeV (n=19*)					
Clinical characteristics (n=6)	Systematic reviews (n=1) Case series/reports (n=5)	Australia (n=6); Bangladesh (n=1); India (n=1); Malaysia (n=1); Philippines (n=1); Singapore (n=1)	Total: 12 Cases: 12	Adults (n=5)	Signs and symptoms (n=4), CFR (n=3) Biochemical (n=3); asymptomatic inf. (n=1) Clinical features to distinguish disease (n=1) Clinical features to assess severity (n=3); long-term complications (n=1)
Transmission and prevention (n=3)	Systematic reviews (n=1) Case series/reports (n=2)	Australia (n=3); Bangladesh (n=1) India (n=1); Malaysia (n=1) Philippines (n=1); Singapore(n=1)	Total: 4 Cases: 4	Adults (n=2)	Incubation period (n=1); transmission routes (n=2); infective body fluids (n=2)
Therapeutics (n=1)	Phase I trial (n=1)	Australia (n=1)	Total: 40 Cases: 0	Adults (n=1)	RCT for m102.4 antibodies (n=1)
Social sciences (n=11)	Systematic reviews (n=1) Interventional (n=1) Cross-sectional (n=9)	Australia (n=11)	Total: 5157 Cases: 0	Older children (n=2) Adults (n=11) Elderly (n=3)	Knowledge/attitudes of vaccines (n=4), diagnostics (n=2), disease (n=3), PPE (n=2); uptake of vaccines (n=2), PPE (n=3); adherence to vaccines (n=1), PPE (n=3)
*LayV (n=2)*					
Clinical characteristics (n=2)	Cross-sectional (n=1) Case series/reports (n=1)	China (n=2)	Total: 61 Cases: 52	Older children (n=1) Adults (n=1) Elderly (n=1)	Signs and symptoms (n=2)
Transmission and prevention (n=1)	Case series/reports (n=1)	China (n=1)	Total: 35 Cases: 26	Older children (n=1) Adults (n=1) Elderly (n=1)	Transmission routes (n=1)

* Some studies took place in multiple countries and/or focused on more than one research domain.

† Laboratory or clinically probable/confirmed cases.

‡ Neonates (≤28 days), infants (29 days to 12 months), young children (1–5 years), older children (6–17 years), adults (18–64 years) and elderly (≥65 years). Some studies did not report on population groups.

IV, intravenous; JEV, Japanese encephalitis virus; PPE, personal protective equipment; RCT, randomised controlled trial.

Most studies focused on NiV (82.1%, 87/106), followed by HeV (17.9%, 19/106) and LayV (1.9%, 2/106). Two studies focused on both NiV and HeV.^5 39^

**Figure 2:**
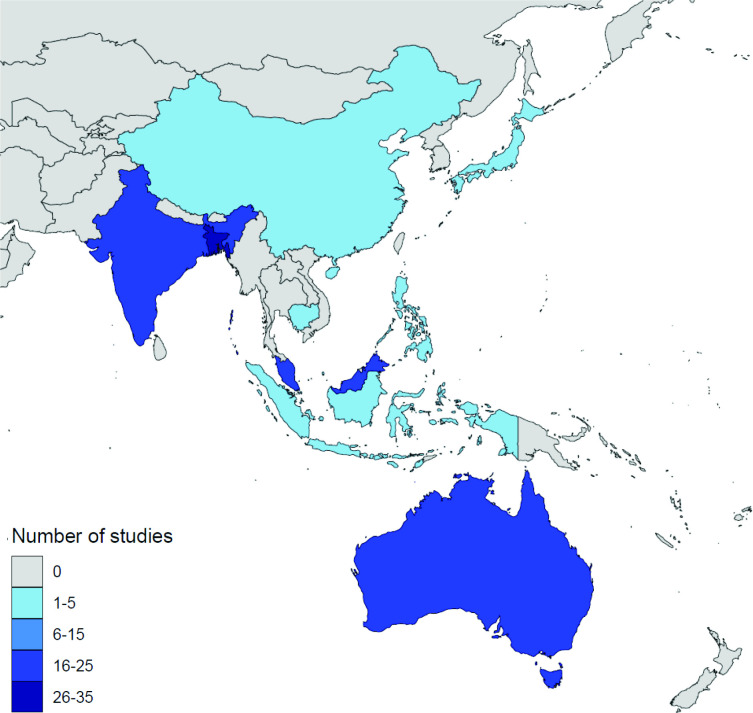
Henipavirus studies by country setting. Map depicting the number of studies on henipaviruses by country. Some studies took place in multiple countries. One study was set outside of this region; a study focused on diagnostics set in Germany.^47^

The studies were set in Asia (82.1%, 87/106), Australia (17.9%, 19/106) and Europe (0.9%, 1/106), six in multiple countries or unspecified (figure 2). All HeV studies were set in Australia (100%, 19/19). Most NiV studies were set in Bangladesh (42.5%, 37/87), followed by Malaysia (24.1%, 21/87), India (29.9%, 26/87), Singapore (12.6%, 11/87), Philippines (3.4%, 3/87), Australia (2.3%, 2/87) and 1.1% (1/87) each in Cambodia, Germany, Indonesia, Japan and Sri Lanka. The two LayV studies were set in China.

**Figure 3:**
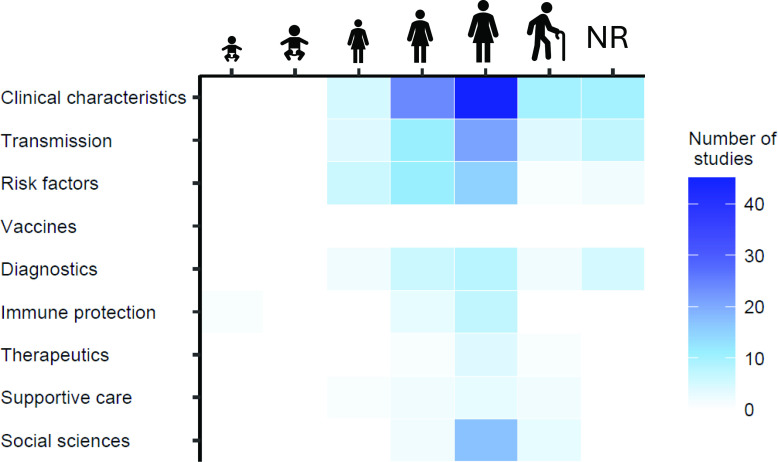
Number of included studies by age group and domain. Pictogram key: neonates (≤28 days), infants (29 days to 12 months), young children (1–5 years), older children (6–17 years), adults (18–64 years), elderly (≥65 years) and not reported (NR). NB: some studies included more than one age group.

Most (76.4%, 81/106) studies included adults, 32.1% (34/106) children, in 22.6% (24/106) age was not reported. Of the studies including children, 0.9% (1/106) included neonates, 6.6% (7/106) young children and 31.1% (33/106) older children (figure 3). The studies included general populations (65.1%, 69/106), animal workers/farmers/forest workers (24.5%, 26/106) and/or healthcare workers (7.5%, 8/106).

### Clinical characteristics

We identified 60 studies presenting data on clinical characteristics (table 1). Of these, 88.3% (53/60) focused on NiV including 4739 confirmed/probable human cases. 10% (6/60) focused on Hendra including 12 overlapping cases, 3.3% (2/60) on Langya presenting on the same 26 cases. Four studies were SRs, all others observational studies. The SRs described asymptomatic infection, CFRs and long-term outcomes in children surviving encephalitis caused by NiV.

Information on clinical characteristics of Nipah virus disease was available for all age groups over 1 year old, although numbers included were limited for children. Five (9.4%) of the NiV studies presented data on young children, 45.3% (24/53) on older children, 77.4% (41/53) on adults, 17.0% (9/53) on elderly and 24.5% (13/53) unreported age group.

Most NiV studies (66.0%, 35/53) reported on CFR, 47.2% (25/53) on signs and symptoms, 22.6% (12/53) long-term sequelae, 18.9% (10/53) laboratory features, 15.1% (8/53) asymptomatic infection, 5.7% (3/53) symptom biomarkers for severe disease, 7.5% (4/53) virus strains and 1.9% (1/53) clinical differential features. One SR identified asymptomatic infection for the NiV-M strain in 0.5% (14/2881) of cases and 1.1% (3/297) asymptomatic infection for the NiV-I strain.^5^

Of the six studies focused on HeV, one was an SR and five case series. Four studies described clinical signs and symptoms, three laboratory features. The SR stated that asymptomatic HeV infection has not been investigated.^5^ Two studies described distinguishing diagnostic features on MRI. One of these described vasculitis in acute HeV encephalitis,^40^ the other study reported that HeV encephalitis patients (n=3) had more grey matter lesions early in the disease process, in contrast to more white matter lesions in NiV encephalitis patients.^41^ Moreover, involvement of white matter necrosis was a sign of disease progression and associated with severe disease.^41^ Three HeV studies including 11 patients reported mortality rates of 50%^5 19^ to 66% in adults.^41^ One study presented neurological sequelae following HeV.^41^

Human LayV infection was characterised in two case series set in China from 2019 to 2021 including 26 cases of LayV only in students, farmers and factory workers.^24 42^ We did not find any records identifying LayV infection in young children. The most common symptoms reported were fever, fatigue, cough and myalgia followed by gastrointestinal symptoms, and in a few cases thrombocytopenia, leucopenia, impaired liver and kidney functions.^24 42^ No deaths were reported, but 53.8% (14/26) were hospitalised.^42^

### Transmission

Transmission was studied in 26.2% (28/106) of the studies, of which 89.3% (25/28) focused on NiV including 2560 cases, 10.7% (3/28) on HeV including four cases and 3.6% (1/28) on LayV disease including 26 cases.

Nipah virus transmission was described in three SRs, covering incubation period, human-to-human routes of transmission and infectivity of body fluids. The primary studies were 8.0% (2/25) cohort, 28.0% (7/25) case–control, 32.0% (8/25) cross-sectional studies and 20.0% (5/25) case series/reports. Populations included were young children (16.0%, 4/25), older children (40.0%, 10/25), adults (72.0%, 18/25) and elderly (12.0%, 3/25). Of the NiV studies, 36.0% (9/25) described incubation period, which ranged from 6 to 16 days, with a span of 4 days to 2 months reported.

16 articles (64.0%, 16/25) presented information on transmission routes: 50.0% (8/16) explored transmission via close human-to-human contact, 31.3% (5/16) body secretions, 18.8% (3/16) did not identify transmission through human contact and 18.8% (3/16) in healthcare settings. Body fluids that tested positive for NiV RNA were throat swabs,^43 44^ nasal swabs,^43 44^ urine^43^ and semen.^5 45^ Two studies reported that UV treatment with or without thermal heating was able to effectively inactivate NiV in serum and plasma.^46 47^

HeV transmission was only studied in two adults case series and one SR. The SR reported that HeV had an incubation period of 7.5 (5–16) days.^5^ One case series did not identify any cases among 64 contacts (healthcare workers) exposed to two Hendra patients.^19^ One study on shedding detected viral RNA during a 1-month period during the acute phase in one of the two patients.^3^ They did not identify any evidence of prolonged shedding in blood, urine or cerebrospinal fluid samples from these patients during follow-up at 2 and 6 years.

Evidence for LayV transmission was limited. Of 26 cases, 85% were farmers. Contact tracing of 9 patients with 15 close-contact family members did not identify close-contact LayV transmission. No common exposure history among the patients was found for LayV transmission in China.^24^

None of the studies reported on risk of transmission of asymptomatic infection nor on effectiveness of personal protective equipment (PPE) and one on infection prevention control (IPC).

### Risk factors

16 studies, all on NiV, including 4347 cases studied risk factors. Of these, one was an SR including 2156 cases, two cohort studies, five case–control studies, five cross-sectional studies and two case reports. In a cross-sectional study from a village in Bangladesh in 2003 including 12 cases, the median patient age was 12 years (range 4–42 years), demonstrating that children and adults are susceptible to NiV infection.^48^

The SR reported increased risk of NiV encephalitis for males (OR=1.5 (1.1 to 2.0)).^2^ Other reported risk factors for infection were raw date palm sap consumption and harvesting, close contact with an infected person and contact with pigs. Three studies identified proximity of bats^2 49 50^ as a significant risk factor, yet two other studies found it non-significant.^48 51^ Three articles (18.8%, 3/16) identified tachycardia,^52 53^ hypertension^43 52^ and drowsiness at admission^43^ as risk factors for severe disease. Age was identified as a risk factor for mortality; one study reported that patients who died were significantly older (median 28 years; range 2–60 years) compared with survivors (median 15 years; range 4–50 years; p=0.018).^54^ None of the studies explored risk factors for long-term complications.

### Diagnostics

14 studies, all focused on NiV, explored diagnostics, with a total of 714 positive samples included. Tests included real-time PCR (n=1),^55^ qRT-PCR (n=1),^56^ a portable PCR analyser (n=1),^57^ ELISA (n=10) and a split NanoLuc biosensor (n=1).^58^ Human samples used for testing were serum (85.7%, 12/14), throat swabs (21.4%, 3/14), cerebrospinal fluid (7.1%, 3/14), blood (14.3%, 2/14), rectal swabs (7.1%, 1/14) and urine (7.1%, 2/14). The study focused on a qRT-PCR assay detected NiV RNA in urine, bronchial wash and cerebrospinal fluid.^56^

Sensitivity and specificity data were reported in 25% (4/14) of the studies. Studies on an anti-NiV IgM and IgG ELISA reported specificity of 99.3% and sensitivity of 100%.^59 60^ The sensitivity for the split NanoLuc biosensor was slightly lower, with a sensitivity of 98.6% and specificity of 100% for IgG detection.^58^ The portable, battery-operated TrueNat NiV point-of-care (POC) PCR test had a sensitivity of 97% and specificity of 100% compared with the TaqMan real-time RT-PCR.^57^ None of the studies included strain information. Two POC diagnostic tests were identified; the split NanoLuc biosensor^58^ and TrueNat NiV system which could deliver results within an hour.^56^ No multiplex diagnostics for detection of multiple henipaviruses were identified.

### Prophylaxis

We did not identify any studies focused on human vaccines or other pre- or post-exposure prophylaxis for any of the henipaviruses.

### Immune protection

Nine studies (four cross-sectional studies, one case–control, one cohort and three case series/reports) all focused on NiV were identified including a total of 157 cases. Seven studies provided data on seroprevalence, of which three followed up participants for 1–2 years. Four seroprevalence studies included children. One study set in Malaysia reported a seroprevalence of 15.7% (13/83) in adults compared with 5.5% (4/75) in children under 12 years old.^61^ One cross-sectional study (n=92, range: 2–60 years old) identified positive IgG 2 years after the infection and decreased IgM after 2–3 months.^54^ A case–control study including three patients (9–45 years old) infected with NiV identified similar titres of IgM, but higher initial titres of IgG up to 138 days after infection during the 2023 Kerala outbreak.^62^ A case report on a 12-year-old child identified elevated levels of IgG 12 months after illness onset.^63^ A cohort study including 25 survivors reported detectable NiV antibodies and memory B cells 25 years after the 1998 Malaysia outbreak.^64^ One case report identified vertical transfer of NiV immunity in a neonate conceived 1.5 years after the mother was infected.^65^ The baby tested negative for anti-Nipah IgM and PCR and had a high titre of anti-Nipah IgG.

### Therapeutics

Four studies, all focused on NiV reported on therapeutics, two also on HeV (table 2). A phase I RCT tested the safety of m102.4 monoclonal antibodies (mAb) against NiV and HeV in 40 healthy adults, and reported that a single dose was safe.^39^ A non-randomised interventional study, including 140 patients with Nipah encephalitis, reported significantly higher survival ratio in patients treated with ribavirin compared with controls who had not been administered the drug due to non-availability or refusal (32% (45/140) vs 54% (29/54), p=0.011).^66^ However, patients receiving ribavirin were ventilated for longer and stayed longer in the hospital than controls (9.4±8.7 days vs 4.2±2.4 days, p<0.001). In addition, the sample size was small, no blinding and controls unmatched.^66^ The only study including children and older adults was a cross-sectional study including 94 patients aged 13–68 years old. The authors did not identify any significant effects on survival in those administered ribavirin.^17^ A case series provided data on intravenous aciclovir administration in nine patients with NiV infection, where 88.9% (8/9) survived, but the sample size was small, the study uncontrolled and several patients deteriorated before recovering, thus the effect of acyclovir therapy is not clear.^67^

**Table 2:** Summary of key findings on therapeutics

Treatment	Virus	Outcome measure	Number of studies, country, population	Results
m102.4 antibody^39^	NiV and HeV	Safety	Phase I RCT, Australia, n=40 healthy adults	No adverse events, differences in haematological or coagulation parameters observed after administration to healthy adults.
Ribavirin^66^	NiV	Mortality	Non-randomised open-label trial, Malaysia, n=140 patients	The study reported a 36% reduction in mortality in patients with acute Nipah encephalitis (32% (45/140) vs 54% (29/54), p=0.011) in untreated patients. Ribavirin patients were ventilated longer and had longer hospital stay.
Ribavirin^17^	NiV	Mortality	Cross-sectional study, Malaysia, n=94 patients, 13–86 years old	The study noted no significant effects of ribavirin administration on survival.
Aciclovir^67^	NiV	Mortality	Case series, Singapore n=9 patients 24–66 years old	8/9 patients survived, several deteriorated before recovery.

HeV, Hendra virus; NiV, Nipah virus; RCT, randomised controlled trial.

### Supportive care

None of the identified studies focused on supportive care, though three NiV studies set in Malaysia mentioned administration of supportive care. One cohort study (n=103, 4–75 years old) reported that 62 patients were ventilated.^52^ A cross-sectional study including 94 patients described different types of supportive care, such as intravenous phenytoin to control seizures.^17^ In addition, aspirin and pentoxifylline were administered to most patients for vasculitis-induced thrombosis, and half of the 94 patients required mechanical ventilatory support.^17^ Another case study reported need for ventilation in two out of three patients, of which one was also intubated and got administered fluids.^68^ However, no information was presented on the effectiveness of supportive care on recovery time, length of hospital stays, complications, mortality or risk of side effects. This shows a gap in implementation of comparative studies with capacity to identify optimal supportive care.

### Social and behavioural research

Out of 17 studies identified, most (64.7%, 11/17) focused on Hendra including 5157 participants in total, 35.3% (6/17) on Nipah disease, including 4921 participants, none on Langya disease. All Hendra studies were set in Australia, four Nipah studies in Bangladesh and two in India. 5.9% (1/17) were SRs, 11.8% (2/17) non-randomised interventional (before-and-after studies) and 82.4% (14/17) cross-sectional studies.

All Hendra studies included adults, two also included older children.^69 70^ The majority (90.9%, 10/11) included animal workers (mainly veterinarians) and horse owners, one focused on the general public though the survey was shared by horse industry and wildlife interest groups. The SR was focused on communication interventions and factors driving the uptake of HeV vaccines for horses amongst veterinarians and horse owners.^71^ One study including inhabitants in Queensland explored attitudes to flying foxes to inform bats management decisions in urban environments, concluding that a more targeted management approach was needed.^70^ In another study, three citizens’ juries were tasked with considering approaches to manage HeV risk in Australia, where the majority voted that veterinarians should not be obliged to attend unvaccinated horses.^72^ Knowledge or attitudes towards HeV vaccines for horses were reported in 28.6% (4/11) of articles, of which two also reported knowledge or attitudes towards diagnostics.^72 73^ Knowledge of the disease was explored in 21.4% (3/11) and attitudes towards PPE in 14.3%.^74 75^

Furthermore, two reported on uptake of vaccines^69 71^ and three on uptake and adherence of PPE.^76^,^78^ One study covered risk communication of veterinarians with horse owners.^74 76 77^

Of the six NiV studies, all included adults. Five presented findings on knowledge about the disease and transmission risks. Two focused on raw sap consumption habits and community perceptions. Two studies assessed effects of infection prevention training in healthcare workers^79^ or self-instruction training among the general population in identifying and managing NiV,^80^ but did not elaborate on the training specifics. One study assessed psychological well-being of hospital staff during Nipah outbreaks in India.^81^ Four NiV social and behavioural research studies focused on the general population, two included healthcare workers.^79 81^

We did not identify any studies focusing on Langya disease, nor on access, uptake or adherence to clinical management guidelines, or recommended care for either Nipah or Hendra.

## Discussion

Our data highlight the limited evidence available for henipaviruses in both quantity and quality, despite Nipah virus circulating since 1998 in regions in Asia with a high mortality rate. There are concerning gaps in the evidence, in particular on disease understanding and medical countermeasures. High-quality study designs are scarce and available studies are of limited scope, particularly there is a lack of inclusion of at-risk populations such as infants and children.

We identified limited high-quality evidence on henipavirus treatments and prophylaxis, with a notable lack of published randomised controlled trials. Evidence on henipaviruses is likely limited for three reasons: epidemiological challenges due to low prevalence and unpredictable outbreaks, operational challenges in outbreak areas with little research infrastructure and clinical research capacity, and challenges in the commercial market for NiV therapeutics due to limited financial resources.^82^ The relatively small case numbers in the few interventional studies identified, unmatched controls and limited data on disease severity and timing of administration result in limited statistical power, reducing the strength and generalisability of the findings. This is reflected in the conflicting results on benefits of ribavirin in patients with Nipah virus infection.^17 66^ It likely reflects the challenges in EID research, and where observational studies are important for the early response to new and emerging infectious diseases, for example, to characterise illness and identify people at risk of severe disease, for triage to critical care monitoring, and for shielding in the community. However, to identify effective treatment and prophylaxis, there is a need to prepare for implementation of well-designed adaptive medical countermeasure trial protocols in high-risk areas. The m102.4 mAb candidate developed for NiV and HeV infection showed good safety records in a phase I trial,^39^ and a SR by Chan et al,^83^ presents an in-depth overview of additional Nipah drug candidates for such trials.

Analysis of global research funding allocation shows that more than 80% of the funding allocated to Nipah virus disease is allocated to three categories.^84^ Of the grants analysed, most were allocated to pathogen, transmission and diagnostic studies (n=60), therapeutic research and development (n=40) and vaccine research and development (n=24). The highest financial commitment ($160.42 million) is on vaccine research, development and implementation. No grants were identified for categories such as infection prevention and control, research informing ethical issues and secondary impacts of disease, response and control measures. Despite this and vaccines listed as a key priority in the Nipah Research & Development (R&D) roadmap in 2019, we did not identify any vaccine or post-exposure prophylaxis studies.^85^ This may be due to a combination of factors: a need to match resources to roadmaps, the COVID-19 pandemic restrictions, and the challenges of implementing EID research.^32^ However, there are new developments on the horizon, including a phase II ChAdOx1 NiV-B Nipah vaccine trial which has been granted support from the priority medicines scheme of the European Medicines Agency.^86^ The trial was launched in Bangladesh in December 2025. Further strategies recommended in the R&D roadmap were development of vaccines that cover multiple henipaviruses, or Nipah and measles, a related paramyxovirus and public health priority.^85^ Lacking effective prophylaxis and therapeutics, access to evidence-based optimal supportive care is fundamental for improving survival rates.^87^ Optimal supportive care guidance will not only benefit patients, but also prioritise healthcare investments, especially relevant for resource-limited settings.^88^

Studies on disease perceptions and attitudes towards vaccination, treatment, uptake of diagnostics and care seeking behaviour are vital for managing outbreak-prone viruses. These insights cannot only inform risk communication and public engagement, but also lay the groundwork for future deployment of countermeasures, especially important in contexts where trust, misinformation and health system capacity pose significant challenges.^89^ Our research shows a clear evidence gap in human-focused social and behavioural research, with existing evidence focusing mainly on human attitudes towards HeV vaccination in horses. Vaccination of intermediate hosts can reduce the chance of spillover events and mitigate economic consequences.^90^ However, as most pigs are used as food source rather than for recreation or transport, attitudes towards vaccination of pigs may be very different. While the gathered evidence on HeV from Australian settings is useful, Asian perspectives on vaccination against NiV are crucial. Knowledge of such attitudes is helpful in applying a One Health approach for henipavirus control. We did not identify evidence on the effectiveness of prevention and control methods, which is a challenge for effective resource prioritisation and to mitigate wider health and socio-economic consequences, particularly relevant to resource-limited settings.^91^ We need to move beyond short-term fixes and build systems that truly connect human, animal and environmental health, so future outbreaks can be detected early and managed effectively.

Although clinical characteristics for NiV and HeV infections have been described, an incomplete understanding of disease progression and transmission remains.^92^ More information on biomarkers for differential diagnoses and to grade severity of the disease is needed, especially for younger and older populations (online supplemental appendix 4). Findings show that there is a risk of human-to-human transmission for Nipah virus, yet there were no studies exploring risk of infection from different body fluids, nor risk of reinfection following natural infection.^45^

The initial Nipah outbreak was caused by NiV-M, followed by outbreaks in Bangladesh and India caused by NiV-B or the more recently identified Indian strain (NiV-I). Strain-to-strain differences in transmission and pathology of NiV have been suggested,^12 93^ although dissecting these from other factors like healthcare and containment strategies is challenging. Diagnostics that distinguish different strains are needed to determine strain-specific differences in transmission. Timely diagnosis is needed to establish intermediary species in outbreaks. For example, in 2014 an outbreak in the Philippines with a henipaviral illness in 17 people was linked to NiV by RT-PCR and NGS with up to 99% similarity. However, this was based on one patient sample only taken late during the outbreak and the quality of the reads was relatively poor.^94^ Of note, horses were identified as an intermediate species in this outbreak, calling into question the linkage with NiV. Thus, there was a lost opportunity to understand the outbreak, underscoring the need for improved diagnostic capability, including rapid diagnostics for field testing, and collaboration with One Health organisations, as recommended in the South-East Asia Regional Roadmap for Diagnostic Preparedness. The roadmap advocates for agile and resilient laboratory policies and systems, research into new technologies, using a One Health approach to surveillance, diagnosis and control of NiV in humans, animals and wildlife.^95^

It is important to acknowledge the limitations of this study. First, although evidence gaps were identified by the absence of articles and limited inclusion of diverse population groups and coverage by study setting, the presence of studies identified does not necessarily indicate that the gap is fully addressed, due to study limitations. Many studies were of lower level of evidence and with heterogenous reporting which limits capacity for statistical analysis. The interventional studies included small sample sizes and unmatched controls, with associated risk of bias and confounding. Second, as part of the RRNA protocol, we do not perform a risk of bias assessment, but appraise studies by scope, study design, data reported, and inclusivity. Furthermore, articles cited in SRs were not deduplicated from our search results, as per our RRNA protocol, which means potential overreporting of case numbers, which prevents further statistical analysis, beyond descriptive analysis and presentation of the findings reported. Nevertheless, our findings present a comprehensive overview of all published peer-reviewed evidence on henipaviruses detected to cause disease in humans, focused on the research domain topics. This rich information can inform interim strategies including research prioritisation, investment and coordination to forward our knowledge into henipaviruses.

The strengths of this RRNA lie in its comprehensive search and wide scope, which enabled us to identify relevant evidence gaps for clinical and public health management. Furthermore, focusing on a viral genus, rather than a single disease, in line with WHO's latest R&D priority pathogen strategy,^96^ enabled us to identify the emerging Langya virus detected to cause disease in humans. The evidence on LayV is sparse, and it remains an ill-defined species with no isolates available,^97^ but illustrates the potential for yet undetected henipaviruses to infect humans, and justifies the approach to focus on viral families and a syndromic approach for study protocols and trial candidates, where feasible.

These findings highlight multiple priorities for further research. Notably, there is a clear need to identify effective therapeutics, prophylaxis and supportive care for all risk-population groups, to inform standardised evidence-based guidelines and improve equity in access to evidence-based prevention, care and control. Though Nipah is the main priority pathogen, a holistic, viral family and one health approach, engaging clinicians, veterinarians, farmers, microbiologists, environmental and technological experts, is recommended, combined with efforts to strengthen preparedness for the early detection of new, and emerging henipaviruses and variants in risk regions. Likewise, social and behavioural studies can adopt a One Health henipavirus approach, but for these studies, it is vital to engage diverse population groups and in different settings. Attitudes to vaccines can vary by disease and within population groups in the same area,^98^ hence engaging representative community members is key. The unpredictable nature of henipavirus outbreaks asks for ready-to-launch, pre-approved and pre-positioned patient-centred, observational characterisation studies and adaptive trials to be implemented at the onset of an outbreak to strengthen capacity to include sufficient number of cases across demographics and disease severity to generate meaningful results.^99^

## Conclusions

This RRNA shows a need to invest in research into medical countermeasures and diverse cohorts including children and elderly to address the evidence gaps identified, to protect populations in endemic regions. Priorities should be targeted towards Nipah, but ideally in a paramyxovirus family approach, to strengthen preparedness to emergence of new henipaviruses and variants. Considering the sporadic outbreaks and case numbers, a combination pre-positioned research strategy including epidemiological, observational and adaptive trial protocols are recommended, to characterise pathogenesis, immune response and identify optimal care, prevention and control strategies. Engagement with communities, co-design and co-production of studies are recommended to facilitate implementation and uptake of research outputs. Furthermore, there is a need to review capacity to detect and manage emerging Nipah cases in neighbouring regions for early detection and community protection.

## Supplementary material

10.1136/bmjph-2025-004195 online supplemental file 1

## Data Availability

Data are available in a public, open access repository.
