# Supplementary material for: Henipavirus evidence gaps: a Rapid Research Needs Appraisal
Source: BMJ Public Health. 2026 Jul 6;4(3):e004195. doi: 10.1136/bmjph-2025-004195 (PMC13343017; doi:10.1136/bmjph-2025-004195)
Supplement: online supplemental file 1 [file bmjph-4-3-s001.docx]

# Appendix 1-4

# Appendix 1 Search strategy

## 1.1 General search strategy

University of Oxford/ Nuffield Department of Medicine/ Pandemic Sciences Institute

**Search report: 06 October 2023**

## Rapid Research Needs Appraisal (RRNA) for Nipah/Hendra

Eli Harriss (Bodleian Health Care Libraries, University of Oxford, ORCID: 0000-0003-4635-8959) adapted and translated the searches on 06/10/2023 and managed and deduplicated the results.

## Search Results

| PubMed | 1715 |
| --- | --- |
| Ovid Embase | 1924 |
| Cochrane Database of Systematic Reviews | 0 |
| Cochrane CENTRAL | 13 |
| Epistemonikos | 249 |
| Clinicaltrials.gov | 4 |
| Total | 3905 |
| Total after deduplication | 2380 |
| Additional records from web searches |  |

## Search Strategies

**PubMed: from inception to the search date**

(("Henipavirus"[Mesh]) OR "Henipavirus Infections"[Mesh]) OR (nipah*[Text Word] OR hendra[Text Word] OR NiVE[Text Word])

**Database: Embase 1974 to present**

Link to search history: <https://ovidsp.ovid.com/ovidweb.cgi?T=JS&NEWS=N&PAGE=main&SHAREDSEARCHID=5LFFY8Mtkp0DZOgApBBed6hiibxmF17OWrBgrBT7FKJsALme4NDRTyTHj8nLinGnI>
**Search Strategy:**
**1** exp henipavirus/ (1781)
**2** exp Henipavirus infection/ (759)
**3** (nipah* or hendra or NiVE).ti,ab,kw. (1888)
**4** 1 or 2 or 3 (2448)
**5** symptom/ (165082)
**6** (incubation or symptom* or "natural history" or "clinical feature*" or severity).ti,ab. (3228256)
**7** virus transmission/ (87277)
**8** (transmission or infectiousness).ti,ab. (506532)
**9** (vaccin* or prophyla* or chemoprophyla* or prevent* or protection).ti,ab. (3199066)
**10** diagnosis/ or virus diagnosis/ (1413258)
**11** (diagnosis or diagnostic or "rapid diagnostic test*" or RDT* or screening or detection).ti,ab. (5165049)
**12** immunity/ (82411)
**13** (immunity or immune* or antibod* or anti-bod* or serology or waning).ti,ab. (2322406)
**14** exp *therapy/ (3698507)
**15** (treatment or management or therapy or drug* or intervention*).ti,ab. (12209060)
**16** ("supportive care" or fluid* or electrolyte* or supplement*).ti,ab. (1346294)
**17** mortality/ (902107)
**18** adverse event/ (76581)
**19** exp *complication/ (329092)
**20** (mortality or death or "adverse events" or "side effect*" or "adverse effect*" or complications or sequela*).ti,ab. (4410882)
**21** (immunocompromise* or "controlled human infection*" or "human challenge" or model*).ti,ab. (4835419)
**22** 5 or 6 or 7 or 8 or 9 or 10 or 11 or 12 or 13 or 14 or 15 or 16 or 17 or 18 or 19 or 20 or 21 (23973012)
**23** 4 and 22 (1924)

**Cochrane Database of Systematic Reviews**

**Issue 10 of 12, October 2023**

**Cochrane Central Register of Controlled Trials**

**Issue 10 of 12, October 2023**

#1 (nipah* or hendra* or henipavirus or NiVE):ti,ab,kw 13

**Epistemonikos** <https://www.epistemonikos.org/en/advanced_search>

(title:(nipah* OR hendra) OR abstract:(nipah* OR hendra))

**Clinicaltrials.gov**

Condition or disease: Nipah

## 1.2 Qualitative studies data search

**Search report: 06 October 2023**

## Rapid Research Needs Appraisal (RRNA) for Nipah/Hendra – Qualitative Research

Eli Harriss (Bodleian Health Care Libraries, University of Oxford, ORCID: 0000-0003-4635-8959) adapted and translated the searches on 06/10/2023 and managed and deduplicated the results.

## Search Results

| PubMed | 34 |
| --- | --- |
| Ovid Embase | 48 |
| Total | 82 |
| Total after deduplication | 50 |

## Search Strategies

**PubMed**

**Qualitative Research search filter reference:** FMD(3S)/CHLA [Qualitative research search filter](http://extranet.santecom.qc.ca/wiki/!biblio3s/doku.php?id=concepts:recherche-qualitative) [2014] [Filters available for MEDLINE via Ovid, PubMed] (Accessed at: <https://extranet.santecom.qc.ca/wiki/!biblio3s/doku.php?id=concepts:recherche-qualitative> on 06/10/2023)

((("Henipavirus"[Mesh]) OR "Henipavirus Infections"[Mesh]) OR (nipah*[Text Word] OR hendra[Text Word] OR NiVE[Text Word])) AND (((("semi-structured"[TIAB] OR semistructured[TIAB] OR unstructured[TIAB] OR informal[TIAB] OR "in-depth"[TIAB] OR indepth[TIAB] OR "face-to-face"[TIAB] OR structured[TIAB] OR guide[TIAB] OR guides[TIAB]) AND (interview*[TIAB] OR discussion*[TIAB] OR questionnaire*[TIAB])) OR ("focus group"[TIAB] OR "focus groups"[TIAB] OR qualitative[TIAB] OR ethnograph*[TIAB] OR fieldwork[TIAB] OR "field work"[TIAB] OR "key informant"[TIAB])) OR "interviews as topic"[Mesh] OR "focus groups"[Mesh] OR narration[Mesh] OR qualitative research[Mesh] OR "personal narratives as topic"[Mesh])

**Database: Embase 1974 to present**

**Qualitative Research search filter reference:** FMD(3S)/CHLA [Qualitative research search filter](http://extranet.santecom.qc.ca/wiki/!biblio3s/doku.php?id=concepts:recherche-qualitative) [2014] [Ovid] (Accessed at: <https://extranet.santecom.qc.ca/wiki/!biblio3s/doku.php?id=concepts:recherche-qualitative> on 06/10/2023)

Link to search history: <https://ovidsp.ovid.com/ovidweb.cgi?T=JS&NEWS=N&PAGE=main&SHAREDSEARCHID=5Fvvcl5h1zbuomMdsWPJ0r7IlaTPL5YQHElpIHW033rJH3UktdTvXMkevoMwvy9Ll>
**Search Strategy:**
**1** exp henipavirus/ or exp Henipavirus infection/ or (nipah* or hendra or NiVE).ti,ab,kw. (2448)
**2** (("semi-structured" or semistructured or unstructured or informal or "in-depth" or indepth or "face-to-face" or structured or guide) adj3 (interview* or discussion* or questionnaire*)).ti,ab. or (focus group* or qualitative or ethnograph* or fieldwork or "field work" or "key informant").tw,kw. or qualitative research/ (610490)
**3** 1 and 2 (48)

## 1.3 Updated search 15 May 2025

The searches from 1.1 and 1.2 were repeated by Marieke de Swart on 15 May 2025. Cochrane databases and Clinicaltrials.gov were not searched because the update did not include systematic reviews or ongoing clinical trials.

Search results

|  | General search | |  | Qualitative search | |  |
| --- | --- | --- | --- | --- | --- | --- |
|  | 6/10/2023 | Update 15/5/2025 | + | 6/10/2023 | Update 15/5/2025 | + |
| PubMed | 1715 | 2021 | 306 | 34 | 37 | 3 |
| Ovid Embase | 1924 | 2295 | 371 | 48 | 52 | 4 |
| Cochrane Database of Systematic Reviews | 0 |  |  | NA |  |  |
| Cochrane CENTRAL | 13 |  |  | NA |  |  |
| Epistemonikos | 249 | 249+430 | 430 | NA |  |  |
| Clinicaltrials.gov | 4 |  |  | NA |  |  |
| Total | 3905 | 4746 | 1107 | 82 | 89 | 7 |

# Appendix 2: extraction forms

These extraction forms contain the detailed extractions per disease. General information that was extracted:

- Country
- Study design
- Study dates
- Participants number, sex, ages
- Population groups: Neonates (≤28 days)*, Infants (≤12 months)*, Young children (≤ 5 years)*, Children (18 years old)*, Adults (18 – 64 years old), Elderly (≥65 years old, >75 years old, > 85 years old)*, Pregnant women, Breastfeeding women, Immunosuppressed (by age, medication or illness), Malnourished, Comorbidities and type, Any other specific at-risk, or vulnerable populations specified (e.g. healthcare workers, migrant/Travellers, animal, workers/Farmers/Forestry workers)

Domain-specific questions

| **Domain** | **Question(s)** |
| --- | --- |
| Clinical phenotype and natural history of disease | Which strains are known to cause disease in humans?  What are the signs and symptoms of the disease? (In previously healthy and immunocompromised people)  What are the laboratory (haematology, biochemistry, coagulation etc) features of disease and are they elevated or reduced (no need to extract concentration)?  Are there specific clinical features (signs and symptoms) distinguishing the disease from diagnoses?  Are there distinct clinical features amenable to grading/assessing severity?  Does asymptomatic infection occur and what is the rate?  What is the mortality rate?  Are there risks of long term complications and sequelae? If yes, specify.  What is the prevalence/incidence of disease? |
| Transmission | What is the incubation period of the disease?  What are the routes of transmission (human to human (e.g. airborne, droplet, saliva, body contact, vesicles, blood, transplant, urine, semen, conjunctival fluids, breastmilk, stool)?  What are the infective human body fluids? When and how long are they infectious for?  Are there validated methods to effectively treat body fluids to prevent infection (e.g. of blood samples for blood banks, other body samples used for safe diagnostics)? |
| Prevention | -Is there an effective vaccine? Is it effective against all known strains?  -How many doses are needed for full protection, and dose interval?  -How long is the vaccine effective for? (any risk of waning immunity or unknown)  -How effective is vaccination at preventing disease, reducing severity and transmission?  -Are there known side effects of vaccination?  -Is there pre-exposure drug prophylaxis and how effective is it at preventing disease?  -Is there an effective post-exposure drug prophylaxis and how effective is it at preventing disease?  -Are the prophylaxis well tolerated, what were the proportions completing the course? |
| Diagnostics | -Are there validated diagnostic tests available(what types of tests (e.g. RT-PCR, Elisa, viral culture, etc.)?  -Can they detect all known strains?  - How long does it take to gain results?  - Are there rapid diagnostic tests available?  -Which body fluids are the tests developed/validated for?  -What is the sensitivity and specificity of the different diagnostic tests?  -Are there any identified biomarkers to identify disease and risk of severe disease (extract type of marker– not concentrations)? |
| Immune response | Dose natural infection offer protection against future infections?  What is the antibody response over time from natural infection/what is the rate of waning?  Does vaccination protect against infection, disease and transmission? Does the protection wane over time/what is the rate of waning?  What is the antibody response over time from vaccination? |
| Drug therapy effect | Is there an effective drug?  If yes, how can it be administered? E.g. oral, IV  If yes, what is the effect of drug therapy on:   - recovery time? - length of hospital stay? - risk of complications? - risk of severe disease? - mortality rate?   What are side effects of drug therapy? |
| Supportive care (e.g., electrolytes, fluids) | Is optimal supportive care treatments known?  If yes, what does it entail (e.g. fluids, oxygen, blood transfusion, dialysis, high level/low level ICU care)?  What is the effect of supportive care on:   - recovery time? - length of hospital stay? - complications? - mortality rate?   Are there known side effects or risks of supportive therapy?  Does optimal supportive care require specialist delivery systems, or high level ICU settings? |
| Risk factors | What are the risk factors for infection (e.g. age, gender, ethnicity, smoking, immunosuppression, other comorbidities)?  What are the risk factors for severe disease?  What are the risk factors for acute complications?  What are the risk factors for long term complications/sequela?  What are the risk factors for mortality? |
| Social and behavioural science | Only extract data from primary research studies where these questions are part of the main objectives of the study. This includes studies focused on evaluating the below and those focused on exploring factors that impact on the below (e.g., stigma, trust, engagement, information, socioeconomic etc.)  **Does the study focus on and present data on:**   - **Knowledge of** (dropdown list: the disease, diagnostics, treatments, vaccines, prophylaxis, risk factors, personal protective measures, research). - **Attitude to** (dropdown list: the disease, diagnostics, treatments, vaccines, prophylaxis, risk factors, personal protective measures, participating in research). - **Access to** (Dropdown list: clinical management guidelines, care, diagnostics, information, treatments, vaccines, prophylaxis, personal protective equipment, research) - **Uptake of (**dropdown list: healthcare, diagnostics, treatments, vaccines, prophylaxis, PPE, research studies) - **Adherence to** (dropdown: clinical management guidelines, recommended care, treatments, vaccines, prophylaxis, PPE, research study protocol) - **Are there studies exploring healthcare staff** (dropdown list: training in identifying or managing the disease, capacity or capability in managing the disease, and retention during outbreaks) |

# Appendix 3: list of included studies without data-extraction

Bibliography of case reports/case series with less than ten cases in alphabetical order that were deprioritised for extraction.

| Chow, V. T. K.,Tambyah, P. A.,Yeo, W. M.,Phoon, M. C.,Howe, J.. Diagnosis of Nipah virus encephalitis by electron microscopy of cerebrospinal fluid. Journal of Clinical Virology. 2000. 19:143-147 |
| --- |
| Kumar, A. S. A.,Sohanlal, T.,Prasad, G.,Gupta, M.,Gopal, A.. Single-center experience and lessons learnt from management of NIPAH virus outbreak in India. Open Forum Infectious Diseases. 2019. 6:S659-S660 |
| Lee, K. E.,Umapathi, T.,Tan, C. B.,Tjia, H. T. L.,Chua, T. S.,Oh, H. M. L.,Fock, K. M.,Kurup, A.,Das, A.,Tan, A. K. Y.,Lee, W. L.. The neurological manifestations of Nipah virus encephalitis, a novel paramyxovirus. Annals of Neurology. 1999. 46:428-432 |
| Lim, C. C.,Sitoh, Y. Y.,Lee, K. E.,Kurup, A.,Hui, F.. Meningoencephalitis caused by a novel paramyxovirus: an advanced MRI case report in an emerging disease. Singapore Med J. 1999. 40:356-8 |
| Maramattom, B. V.,Ram, S. A.,Warrier, A.,Balagopal, A.. 'The Bug Stops Here!' Nipah Encephalitis; Kerala 2019. Neurol India. 2023. 71:819-820 |
| Rahman, M. A.,Hossain, M. J.,Sultana, S.,Homaira, N.,Khan, S. U.,Rahman, M.,Gurley, E. S.,Rollin, P. E.,Lo, M. K.,Comer, J. A.,Lowe, L.,Rota, P. A.,Ksiazek, T. G.,Kenah, E.,Sharker, Y.,Luby, S. P.. Date palm sap linked to Nipah virus outbreak in Bangladesh, 2008. Vector Borne Zoonotic Dis. 2012. 12:65-72 |
| Tambyah, P. A.,Tan, J. H.,Ong, B. K.,Ho, K. H.,Chan, K. P.. First case of Nipah virus encephalitis in Singapore. Intern Med J. 2001. 31:132-3 |
| Thulaseedaran, N. K.,Kumar, K. G. S.,Kumar, J.,Geetha, P.,Jayachandran, N. V.,Kamalasanan, C. G.,Mathew, S.,Pv, S.. A Case Series on the Recent Nipah Epidemic in Kerala. J Assoc Physicians India. 2018. 66:63-67 |
| Warrier, A.. A single case outbreak of Nipah Encephalitis from India in May-June 2019. International Journal of Infectious Diseases. 2020. 101:247 |

# Appendix 4: included and data-extracted studies

| Author | Title | Journal | Year | Disease | Setting | Study design | Inclu-sions (n) | Cases (n) | Gender, females (%) | Age (Mean/ median (range)) in years | pop. Groups** | Domains*** |
| --- | --- | --- | --- | --- | --- | --- | --- | --- | --- | --- | --- | --- |
| Abdullah, S. et al | Late-onset Nipah virus encephalitis 11 years after the initial outbreak: A case report | Neurology Asia | 2012 | Nipah | Malaysia | Case series/reports | 1 | 1 | 100 | 35 | General pop. | 1 |
| Agrawal, R. et al | Bangladesh Sees Spike in Nipah Virus Cases: A matter of public health concern? | New Microbes New Infect | 2023 | Nipah | Bangladesh | Cohort | 325 | 325 | NR | NR | General pop. | 1 |
| Alam, M. G. S. et al | An outbreak of Nipah virus in Thakurgaon, northern Bangladesh, 2019 | International Journal of Infectious Diseases | 2020 | Nipah | Bangladesh | Cross-sectional | 19 | 5 | NR | 57 + NR | General pop. | 1 |
| Amal, N. M. et al | Risk factors for Nipah virus transmission, Port Dickson, Negeri Sembilan, Malaysia: results from a hospital-based case-control study | The Southeast Asian journal of tropical medicine and public health | 2000 | Nipah | Malaysia | Case-control | 69 | 52 | 15 | Range: 30-49 | General pop. | 8 |
| Ambat, A. S. et al | Nipah virus: A review on epidemiological characteristics and outbreaks to inform public health decision making | J Infect Public Health | 2019 | Nipah | Bangladesh, India, Malaysia, Singapore | Systematic review | NR | 575 | NR | NR | General pop. | 2 |
| Annand, E. J. et al | Citizens' juries give verdict on whether private practice veterinarians should attend unvaccinated Hendra virus suspect horses | Aust Vet J | 2020 | Hendra | Australia | Cross-sectional | 31 | NA | NR | NR | General pop.,Animal worker,Other (horse owners) | 9 |
| Anoop Kumar, A. S. et al | Clinico-epidemiological presentations and management of Nipah virus infection during the outbreak in Kozhikode district, Kerala state, India 2023 | Journal of Medical Virology | 2024 | Nipah | India | Case series/reports | 6 | 6 | 0 | Range: 9-45 | General pop. | 1 |
| Anoop Kumar, A. S. et al | Clinico-epidemiological presentations and management of Nipah virus infection during the outbreak in Kozhikode district, Kerala state, India 2023 | Journal of Medical Virology | 2024 | Nipah | India | Case series/reports | 6 | 6 | 0 | Range: 9-45 | Gen pop. | **1** |
| Ariyari, S. et al | The first ever Nipah virus outbreak and the best possible response by a tiny state of India | International Journal of Infectious Diseases | 2019 | Nipah | India | Cross-sectional | 18 | 18 | NR | NR | General pop. | 1 |
| Arunkumar, G. et al | Persistence of Nipah Virus RNA in Semen of Survivor | Clin Infect Dis | 2019 | Nipah | India | Case series/reports | 1 | 1 | 0 | 27 | General pop. | 2 |
| Arunkumar, G. et al | Outbreak Investigation of Nipah Virus Disease in Kerala, India, 2018 | The Journal of infectious diseases | 2019 | Nipah | India | Case series/reports | 23 | 23 | 35 | 17-100 | General pop. | 1 |
| Barrett, R. S. et al | The Hendra virus vaccine: perceptions regarding the role of antibody titre testing | Aust Vet J | 2021 | Hendra | Australia | Cross-sectional | 6 | NA | 83 | Range: 50-70 | Animal worker | 9 |
| Bergeron, E. et al | Streamlined detection of Nipah virus antibodies using a split NanoLuc biosensor | Emerging Microbes and Infections | 2024 | Nipah | Bangladesh | Cross-sectional | 712 | 82 | NR | NR | General pop. | 4 |
| Bergeron, E. et al | Streamlined detection of Nipah virus antibodies using a split NanoLuc biosensor | Emerging Microbes and Infections | 2024 | Nipah | Bangladesh | Cross-sectional studies | 712 | 82 | NR | NR | Gen pop. | 4 |
| Cappelle, J. et al | Nipah virus circulation at human-bat interfaces, Cambodia | Bull World Health Organ | 2020 | Nipah | Cambodia | Cross-sectional | 418 | NA | NR | NR | Animal worker | 4 |
| Chadha, M. S. et al | Nipah virus-associated encephalitis outbreak, Siliguri, India | Emerging Infectious Diseases | 2006 | Nipah | India | Case series/reports | 66 | 66 | 42 | 15+ | General pop. | 1, 4 |
| Chakraborty, A. et al | Evolving epidemiology of Nipah virus infection in Bangladesh: Evidence from outbreaks during 2010-2011 | Epidemiology and Infection | 2016 | Nipah | Bangladesh | Case series/reports | 43 | 43 | 30 | Median: 25 (range 2–55) | General pop. | 8 |
| Chakraborty, A. et al | Difficulties in accessing specialized medical care by encephalitis cases during a Nipah virus outbreak in Bangladesh | American Journal of Tropical Medicine and Hygiene | 2011 | Nipah | Bangladesh | Cross-sectional | 31 | 31 | NR | NR | General pop. | 1 |
| Chan, K. P. et al | A survey of Nipah virus infection among various risk groups in Singapore | Epidemiology and Infection | 2002 | Nipah | Singapore | Cohort | 1469 | 22 | NR | NR (adults) | HCW,Animal worker,Other (many) | 1 |
| Chandni, R. et al | Clinical Manifestations of Nipah Virus-Infected Patients Who Presented to the Emergency Department During an Outbreak in Kerala State in India, May 2018 | Clin Infect Dis | 2020 | Nipah | India | Case series/reports | 12 | 12 | 42 | Range: 19-49 | General pop. | 1, 2 |
| Ching, P. K. G. et al | Outbreak of henipavirus infection, Philippines, 2014 | Emerging Infectious Diseases | 2015 | Nipah | Philippines | Case series/reports | 17 | 17 | 6 | 21-60 | General pop. | 8 |
| Chong, H. T. et al | Nipah encephalitis outbreak in Malaysia, clinical features in patients from Seremban | The Canadian journal of neurological sciences | 2002 | Nipah | Malaysia | Cohort | 103 | 103 | 12 | 27485 | Animal worker | 1, 2, 7, 8 |
| Chong, H. T. et al | Treatment of acute Nipah encephalitis with ribavirin | Annals of Neurology | 2001 | Nipah | Malaysia | Non-randomised controlled | 194 | 140 | 15 | Ribavirin: mean = 37.8 (12.0); Control mean = 38.5 (13.9) | General pop. | 6 |
| Chow, C. et al | Long-Term Outcomes in Children Surviving Tropical Arboviral Encephalitis: A Systematic Review | Journal of Tropical Pediatrics | 2021 | Nipah |  | Systematic review | 21 | 10 | NR | NR | General pop. | 1 |
| Chowdhury, T. et al | Nipah Virus Infection Complicated with Encephalitis and Pneumonia Leading to Fatal Outcome: A Case Report from Bangladesh, January 2024 | Journal of Medicine (Bangladesh) | 2025 | Nipah | India | Case series/reports | 1 | 1 | 0 | 38 | General pop. | 1 |
| Chowdhury, T. et al | Nipah Virus Infection Complicated with Encephalitis and Pneumonia Leading to Fatal Outcome: A Case Report from Bangladesh, January 2024 | Journal of Medicine (Bangladesh) | 2025 | Nipah | India | Case series/reports | 1 | 1 | 0 | 38 | Gen pop. | 1 |
| Chua, K. B. et al | Fatal encephalitis due to Nipah virus among pig-farmers in Malaysia | Lancet | 1999 | Nipah | Malaysia | Case series/reports | 3 | 3 | 0 | 34, 51, 52 | General pop. | 7 |
| Chua, K. B. et al | The presence of nipah virus in respiratory secretions and urine of patients during an outbreak of nipah virus encephalitis in Malaysia | Journal of Infection | 2001 | Nipah | Malaysia | Cross-sectional | 20 | 20 | 20 | Range: 14-57 years | Animal worker | 2, 8 |
| Eickmann, M. et al | Inactivation of three emerging viruses - severe acute respiratory syndrome coronavirus, Crimean-Congo haemorrhagic fever virus and Nipah virus - in platelet concentrates by ultraviolet C light and in plasma by methylene blue plus visible light | Vox Sang | 2020 | Nipah | Germany | Case-control | 2 | NA | NR | 18+ | General pop. | 2 |
| Gayathri, K. et al | An observational study in the setting of Nipah virus outbreak Kerala 2018 | Lung India | 2019 | Nipah | India | Case series/reports | 19 | 19 | NR | NR | General pop. | 1 |
| Goh, K. J. et al | Clinical features of Nipah virus encephalitis among pig farmers in Malaysia | New England Journal of Medicine | 2000 | Nipah | Malaysia | Cross-sectional | 94 | 94 | 18 | 13-68 | Animal worker | 1, 6, 7 |
| Gurley, E. S. et al | Person-to-person transmission of Nipah virus in a Bangladeshi community | Emerging Infectious Diseases | 2007 | Nipah | Bangladesh | Case-control | 204 | 36 | 41 | NR | General pop. | 2 |
| Hanna, J. N. et al | Hendra virus infection in a veterinarian | Med J Aust | 2006 | Hendra | Australia | Case series/reports | 1 | 1 | 100 | NR | Animal worker | 1 |
| Harshani, H. B. C. et al | Advancing public health preparedness: Establishment of Nipah virus molecular diagnostic test at the National Reference Laboratory, Sri Lanka | Asian Pacific Journal of Tropical Medicine | 2024 | Nipah | Sri Lanka | Cross-sectional studies | 3 | 3 | NA | NA | Gen pop. | 4 |
| Harshani, H. B. C. et al | Advancing public health preparedness: Establishment of Nipah virus molecular diagnostic test at the National Reference Laboratory, Sri Lanka | Asian Pacific Journal of Tropical Medicine | 2024 | Nipah | Sri Lanka | Cross-sectional | 3 | 3 | NR | NA | General pop. | 4 |
| Hassan, M. M. et al | Understanding the community perceptions and knowledge of bats and transmission of nipah virus in bangladesh | Animals | 2020 | Nipah | Bangladesh | Cross-sectional | 208 | NA | 17 | 24-55* | General pop. | 9 |
| Hassan, M. Z. et al | Nipah Virus Contamination of Hospital Surfaces during Outbreaks, Bangladesh, 2013-2014 | Emerging Infectious Diseases | 2018 | Nipah | Bangladesh | Cohort studies | 332 | 12 | NR | NR | General pop. | 2 |
| Hassan, M. Z. et al | Nipah Virus Contamination of Hospital Surfaces during Outbreaks, Bangladesh, 2013-2014 | Emerging Infectious Diseases | 2018 | Nipah | Bangladesh | Cohort studies | 332 | 12 | NR | NR | Gen pop. | 2 |
| Hegde, S. et al | Potential for person-to-person transmission of henipaviruses: A systematic review of the literature | medRxiv | 2023 | Hendra+Nipah | Australia, Bangladesh, India, Malaysia, Philippines, Singapore | Systematic review | NR | NR | NR | NR | General pop.,Animal worker | 1, 2 |
| Hegde, S. T. et al | Investigating Rare Risk Factors for Nipah Virus in Bangladesh: 2001–2012 | EcoHealth | 2016 | Nipah | Bangladesh | Case-control | 789 | 157 | 39 | Median: 25 , range 0.5-75 | General pop. | 1, 8 |
| Homaira, N. et al | Nipah virus outbreak with person-to-person transmission in a district of Bangladesh, 2007 | Epidemiology and Infection | 2010 | Nipah | Bangladesh | Case-control | 28 | 7 | 29 | 19-30 (cases) | General pop. | 2 |
| Homaira, N. et al | Cluster of Nipah virus infection, Kushtia District, Bangladesh, 2007 | PLoS One | 2010 | Nipah | Bangladesh | Case-control | 32 | 8 | 75 | Range: 27-55 | General pop. | 1, 2, 8 |
| Hossain, M. J. et al | Clinical presentation of Nipah virus infection in Bangladesh | Clinical Infectious Diseases | 2008 | Nipah | Bangladesh | Cross-sectional | 92 | 92 | 38 | Mean (SD) 27.0 (17.0); range 2- 60 | General pop. | 1, 2, 5, 8 |
| Hsu, V. P. et al | Nipah virus encephalitis reemergence, Bangladesh | Emerging Infectious Diseases | 2004 | Nipah | Bangladesh | Cross-sectional | 108 | 25 | 43 | Mehpur: 4–60; median 38  Naogaon: 4-42, median 12 | General pop. | 1, 8 |
| Islam, T. et al | Knowledge and attitude among Bangladeshi healthcare workers regarding the management and infection prevention and control of Nipah virus | Journal of Virus Eradication | 2024 | Nipah | Bangladesh | Cross-sectional | 455 | 0 | 62 | NR (adults) | General pop., Healthcare worker | 9 |
| Islam, T. et al | Knowledge and attitude among Bangladeshi healthcare workers regarding the management and infection prevention and control of Nipah virus | Journal of Virus Eradication | 2024 | Nipah | Bangladesh | Cross-sectional studies | 455 | 0 | 62 | NR (adults) | Healthcare worker | 9 |
| Joob, B. and Wiwanitkit, V. | Cough in Langya henipavirus and severity of infection | JMS - Journal of Medical Society | 2023 | Langya | China | Cross-sectional studies | 26 | 26 | NR | NR | Gen pop. | 1 |
| Joob, B., Wiwanitkit, V. | Cough in Langya henipavirus and severity of infection | Journal of Medical Society | 2023 | Langya | China | Cross-sectional | 26 | 26 | NR | NR | General pop. | 1 |
| Joshi, J. et al | Possible high risk of transmission of the Nipah virus in South and South East Asia: a review | Trop Med Health | 2023 | Nipah | Bangladesh, India, Malaysia, Singapore | Systematic review | 482 | 355 | NR | NR | General pop. | 2 |
| Kaku, Y. et al | Nipah virus infection | Nihon Rinsho | 2007 | Nipah | Bangladesh | Cross-sectional | 57 | 57 | NR | Range: 2-28 | General pop. | 1 |
| Kenmoe, S. et al | Case fatality rate and risk factors for Nipah virus encephalitis: A systematic review and meta-analysis | J Clin Virol | 2019 | Nipah | Bangladesh, India, Malaysia, Philippines, Singapore | Systematic review | 2156 | 2156 | 0-75 | Median: 32 (IQR22.5-38) | General pop. | 1, 8 |
| Khan, S. A. et al | Major bat-borne zoonotic viral epidemics in Asia and Africa: A systematic review and meta-analysis | Vet Med Sci | 2022 | Nipah | Bangladesh, India, Malaysia, Singapore | Systematic review | 265 | 265 | NR | NR | General pop. | 1 |
| Kropich-Grant, J. N. et al | Communication Interventions and Assessment of Drivers for Hendra Virus Vaccination Uptake | Vaccines | 2023 | Hendra | Australia | Systematic review | 239 | NA | NR | NR | General pop.,Animal worker | 9 |
| Kumar, C. P. G. et al | Infections among contacts of patients with Nipah virus, India | Emerging Infectious Diseases | 2019 | Nipah | India | Cross-sectional | 279 | 3 | 53 | Median: 37 (HCW); Median: 39 (contacts) | HCW,General pop. | 1, 2 |
| Kung, N. et al | Hendra virus and horse owners--risk perception and management | PLoS One | 2013 | Hendra | Australia | Cross-sectional | 1431 | NA | 85 | 16-65 * | Animal worker | 9 |
| Kung, N. Y. et al | Flying-foxes in the Australian urban environment-community attitudes and opinions | One Health | 2015 | Hendra | Australia | Cross-sectional | 2744 | NA | 64 | 16-55 * | General pop. | 9 |
| Lee, K. H. et al | Changing contact patterns over disease progression: Nipah virus as a case study | Journal of Infectious Diseases | 2020 | Nipah | Bangladesh | Case-control | 1718 | 94 | 31 | 38 case patients under 14yo, 21 15-29yo, 23 30-44yo, 12 older than 45 | General pop. | 1 |
| Lim, C. C. et al | Nipah virus encephalitis: serial MR study of an emerging disease | Radiology | 2002 | Nipah | Malaysia, Singapore | Case series/reports | 12 | 11 | 8 | Range: 24-65 | Animal worker | 1 |
| Lim, C. C. et al | Nipah viral encephalitis or Japanese encephalitis? MR findings in a new zoonotic disease | AJNR Am J Neuroradiol | 2000 | Nipah | Malaysia | Cohort | 8 | 8 | 13 | Range: 23-65 | Animal worker | 1 |
| Lim, C. C. et al | Late clinical and magnetic resonance imaging follow up of Nipah virus infection | J Neurol Neurosurg Psychiatry | 2003 | Nipah | Singapore | Cohort | 22 | 22 | 5 | Range: 24-65 (13 symptomatic cases) | Animal worker | 1 |
| Lo, M. K. et al | Characterization of nipah virus from outbreaks in Bangladesh, 2008-2010 | Emerging Infectious Diseases | 2012 | Nipah | Bangladesh | Case series/reports | 21 | 17 | NR | NR | General pop. | 1 |
| Luby, S. P. et al | Recurrent zoonotic transmission of Nipah virus into humans, Bangladesh, 2001-2007 | Emerging Infectious Diseases | 2009 | Nipah | Bangladesh | Case series/reports | 122 | 122 | 39 | 27426 | General pop. | 1, 2, 4 |
| Manyweathers, J. et al | Risk Mitigation of Emerging Zoonoses: Hendra Virus and Non-Vaccinating Horse Owners | Transbound Emerg Dis | 2017 | Hendra | Australia | Cross-sectional | 210 | NA | NR | NR | Other (horse owners) | 9 |
| Mendez, D. et al | Hendra virus in Queensland, Australia, during the winter of 2011: veterinarians on the path to better management strategies | Prev Vet Med | 2014 | Hendra | Australia | Cross-sectional | 200 | NA | 52 | NR | Animal worker | 9 |
| Mendez, D. et al | Response of Australian veterinarians to the announcement of a Hendra virus vaccine becoming available | Australian Veterinary Journal | 2013 | Hendra | Australia | Cross-sectional | 204 | NA | 51 | NR | Animal worker | 9 |
| Mendez, D. H. et al | Difficulties experienced by veterinarians when communicating about emerging zoonotic risks with animal owners: the case of Hendra virus | BMC Vet Res | 2017 | Hendra | Australia | Cross-sectional | 48 | NA | 52 | NR | Animal worker | 9 |
| Mohamed, F. et al | Assessing Psychological Morbidities in Hospital Staff During Nipah Virus Concerns: Prevalence of Depression and Anxiety | The primary care companion for CNS disorders | 2025 | Nipah | India | Cross-sectional studies | 240 | 0 | 73 | Range: 18-60 | HCW | 9 |
| Mohamed, F. et al | Assessing Psychological Morbidities in Hospital Staff During Nipah Virus Concerns: Prevalence of Depression and Anxiety | The primary care companion for CNS disorders | 2025 | Nipah | India | Cross-sectional | 240 | 0 | 73.3 | Range: 18-60 | General pop., Healthcare worker | 9 |
| Mokbul, M. I. et al | Assessment of the general population knowledge about the emergence of Nipah virus outbreak in Bangladesh: A nationwide cross-sectional study | Journal of Virus Eradication | 2025 | Nipah | Bangladesh | Cross-sectional | 2121 | 0 | 48.4 | Adults | General pop. | 9 |
| Mokbul, M. I. et al | Assessment of the general population knowledge about the emergence of Nipah virus outbreak in Bangladesh: A nationwide cross-sectional study | Journal of Virus Eradication | 2025 | Nipah | Bangladesh | Cross-sectional studies | 2121 | 0 | 48 | NR (adults) | Gen pop. | 9 |
| Mounts, A. W. et al | A cohort study of health care workers to assess nosocomial transmissibility of Nipah virus, Malaysia, 1999 | Journal of Infectious Diseases | 2001 | Nipah | Malaysia | Cohort | 84 | 3 | 18 | 13-68 | General pop. | 1 |
| Nahar, N. et al | Raw Sap Consumption Habits and Its Association with Knowledge of Nipah Virus in Two Endemic Districts in Bangladesh | PLoS One | 2015 | Nipah | Bangladesh | Cross-sectional | 1777 | NA | 50 | Mean: 40 | General pop. | 9 |
| Nakka, P. et al | MRI findings in acute Hendra virus meningoencephalitis | Clin Radiol | 2012 | Hendra | Australia | Case series/reports | 3 | 3 | 66.6 | Range: 21-55 | General pop. | 1 |
| Naser, A. M. et al | Integrated cluster- and case-based surveillance for detecting stage III zoonotic pathogens: An example of Nipah virus surveillance in Bangladesh | Epidemiology and Infection | 2015 | Nipah | Bangladesh | Cross-sectional | 176 | 62 | 32 | Median: 21 years (IQR 7–40) | General pop. | 1 |
| Nazneen, A. et al | Nipah virus infection in 2018-19 Nipah season in Bangladesh | American Journal of Tropical Medicine and Hygiene | 2019 | Nipah | Bangladesh | Cross-sectional | 521 | 8 | NR | NR | General pop. | 1 |
| Ng, B. Y. et al | Neuropsychiatric sequelae of Nipah virus encephalitis | Journal of Neuropsychiatry and Clinical Neurosciences | 2004 | Nipah | Singapore | Case series/reports | 9 | 9 | 92 | 24-55 | Animal worker | 1 |
| Nikolay, B. et al | Transmission of nipah virus - 14 years of investigations in Bangladesh | New England Journal of Medicine | 2019 | Nipah | Bangladesh | Cross-sectional | 248 | 248 | 36 | Median: 24 (IQR 10-35) | General pop.,HCW | 1, 2 |
| Nikolay, B. et al | Developing a public health tool to monitor the transmission potential of nipah virus during outbreaks | American Journal of Tropical Medicine and Hygiene | 2018 | Nipah | Bangladesh | Cross-sectional | NR | NA | NR | NR | General pop. | 1, 2 |
| Ong, H. M. et al | Malaysia outbreak survivors retain detectable Nipah antibodies and memory B cells after 25 years | Journal of Infection | 2025 | Nipah | Malaysia | Cohort | 48 | 25 | 20 | Range: 14-64 | General pop. | 5 |
| Ong, H. M. et al | Malaysia outbreak survivors retain detectable Nipah antibodies and memory B cells after 25 years | Journal of Infection | 2025 | Nipah | Malaysia | Cohort studies | 48 | 25 | 20 | Range: 14-64 | Gen pop. | 5 |
| Pallivalappil, B. et al | Dissecting an Outbreak: A Clinico-epidemiological Study of Nipah Virus Infection in Kerala, India, 2018 | J Glob Infect Dis | 2020 | Nipah | India | Case series/reports | 23 | 18 | NR | NR | General pop. | 1 |
| Parashar, U. D. et al | Case-control study of risk factors for human infection with a new zoonotic paramyxovirus, Nipah virus, during a 1998-1999 outbreak of severe encephalitis in Malaysia | J Infect Dis | 2000 | Nipah | Malaysia | Case-control | 110 | 97 | 18 | 28004 | General pop. | 8 |
| Paton, N. I. et al | Outbreak of Nipah-virus infection among abattoir workers in Singapore | Lancet | 1999 | Nipah | Singapore | Case series/reports | 11 | 11 | 0 | 24-66 | Animal worker | 1, 2, 4, 5, 6 |
| Playford, E. G. et al | Human Hendra virus encephalitis associated with equine outbreak, Australia, 2008 | Emerging Infectious Diseases | 2010 | Hendra | Australia | Case series/reports | 2 | 2 | 100 | 21 and 33 | Animal worker | 1 |
| Playford, E. G. et al | Safety, tolerability, pharmacokinetics, and immunogenicity of a human monoclonal antibody targeting the G glycoprotein of henipaviruses in healthy adults: a first-in-human, randomised, controlled, phase 1 study | The lancet. Infectious diseases | 2020 | Hendra+Nipah | Australia | Phase 1 trial | 40 | NA | 40 | 18-44 | General pop. | 6 |
| Ramachandran, R. et al | Sero-prevalence of Nipah antibodies among close contacts of the index case during 2019 Ernakulam outbreak | J Family Med Prim Care | 2022 | Nipah | India | Cross-sectional | 49 | NA | 51 | Mean (SD): 29.4 (12.9) | HCW, General pop. | 1, 2, 5 |
| Sahay, R. R. et al | Comparative immune profiling in survivors of the 2023 Nipah outbreak in Kerala state, India | Journal of Medical Virology | 2024 | Nipah | India | Case-control | 13 | 3 | 0 | Range: 9-45 | General pop. | 1, 5 |
| Sahay, R. R. et al | Encephalitis-predominant Nipah virus outbreaks in Kerala, India during 2024 | Journal of Infection and Public Health | 2025 | Nipah | India | Cross-sectional | 104 | 104 | 0 | Range: 12-35 | General pop., Animal worker/farmer/forestry worker | 1 |
| Sahay, R. R. et al | Comparative immune profiling in survivors of the 2023 Nipah outbreak in Kerala state, India | Journal of Medical Virology | 2024 | Nipah | India | Case-control studies | 13 | 3 | 0% | Range: 9-45 | Gen pop. | 1, 5 |
| Sahay, R. R. et al | Encephalitis-predominant Nipah virus outbreaks in Kerala, India during 2024 | Journal of Infection and Public Health | 2025 | Nipah | India | Cross-sectional studies | 104 | 104 | 0 | Range: 12-35 | Gen pop., Animal worker | 1 |
| Sakharkar, S. et al | Assess the effectiveness of self-instruction module on knowledge regarding nipah virus infection and its prevention among general population | Indian Journal of Forensic Medicine and Toxicology | 2021 | Nipah | India | Non-randomised controlled | 120 | NA | NR | 18-58 | General pop. | 9 |
| Sarji, S. A. et al | MR imaging features of Nipah encephalitis | AJR Am J Roentgenol | 2000 | Nipah | Malaysia | Case series/reports | 31 | 31 | 26 | 15-68 | General pop. | 1 |
| Satter, S. M. et al | Vertical Transfer of Humoral Immunity against Nipah Virus: A Novel Evidence from Bangladesh | Tropical Medicine and Infectious Disease | 2023 | Nipah | Bangladesh | Case series/reports | 3 | 2 | 66 | 30 and 4 (cases), 25 (probable case) | General pop. | 5 |
| Satter, S. M. et al | Tackling a global epidemic threat: Nipah surveillance in Bangladesh, 2006-2021 | PLoS Negl Trop Dis | 2023 | Nipah | Bangladesh | Cross-sectional | 322 | 322 | 38 | Median: 24 (IQR, 10–35) (cases) | General pop. | 1, 2, 4, 8 |
| Sazzad, H. M. et al | Performance of risk exposure screening questions to identify NIPAH cases on admission in surveillance hospitals in Bangladesh | American Journal of Tropical Medicine and Hygiene | 2013 | Nipah | Bangladesh | Cohort | 19 | 19 | NR | NR | Other at risk pop. | 8 |
| Sazzad, H. M. S. et al | Nipah virus infection outbreak with nosocomial and corpse-to-human transmission, Bangladesh | Emerging Infectious Diseases | 2013 | Nipah | Bangladesh | Case-control | 74 | 16 | 44 | 4-60 (cases) | General pop. | 1, 2, 4, 8 |
| Sazzad, H. M. S. et al | Exposure-based screening for nipah virus encephalitis, Bangladesh | Emerging Infectious Diseases | 2015 | Nipah | Bangladesh | Cross-sectional | 328 | 17 | NR | NR | General pop. | 4, 8 |
| Sejvar, J. J. et al | Long-term neurological and functional outcome in Nipah virus infection | Annals of Neurology | 2007 | Nipah | Bangladesh | Case series/reports | 22 | 22 | 45 | 18415 | General pop. | 1 |
| Selvey, L. A. et al | Infection of humans and horses by a newly described morbillivirus | Medical Journal of Australia | 1995 | Hendra | Australia | Case series/reports | 2 | 2 | 0 | 40-49 | Animal worker | 1, 2 |
| Shete, A. M. et al | Development of Nipah virus-specific IgM & IgG ELISA for screening human serum samples | Indian J Med Res | 2022 | Nipah | India | Case-control | 607 | 49 | NR | NR | General pop. | 4 |
| Shete, A. M. et al | Antibody response in symptomatic & asymptomatic Nipah virus cases from Kerala, India | Indian J Med Res | 2021 | Nipah | India | Cross-sectional | 5 | 5 | NR | NR | General pop. | 4, 5 |
| Siva, S. R. et al | Ten year clinical and serological outcomes of Nipah virus infection | Neurology Asia | 2009 | Nipah | Malaysia | Cross-sectional | 70 | 39 | 47 | Mean (SD): 46 (1.8) (cases) | General pop. | 1 |
| Tan, C. T. et al | Relapsed and late-onset Nipah encephalitis | Annals of Neurology | 2002 | Nipah | Malaysia | Cross-sectional | 160 | 22 | 36 | 20333 | General pop. | 1 |
| Taylor, C. et al | No evidence of prolonged Hendra virus shedding by 2 patients, Australia | Emerging Infectious Diseases | 2012 | Hendra | Australia | Case series/reports | 2 | 2 | 100 | Mean: 23 (21-25) | Animal worker | 2 |
| Thiagarajan, K. | Nipah virus: Kerala reports second death in four months | BMJ | 2024 | Nipah | India | Case series/reports | 1 | 1 | 0 | 24 | General pop., Student | 1 |
| Thiagarajan, K. | Nipah virus: Kerala reports second death in four months | BMJ | 2024 | Nipah | India | Case series/reports | 1 | 1 | 0 | 24 | Gen pop., Student | 1 |
| Thomas, B. et al | Nipah Virus Infection in Kozhikode, Kerala, South India, in 2018: Epidemiology of an Outbreak of an Emerging Disease | Indian J Community Med | 2019 | Nipah | India | Cross-sectional | 18 | 18 | 39 | Range: 19-59 | General pop. | 1, 2 |
| Thompson, K. et al | Willingness to adopt personal biosecurity strategies on thoroughbred breeding farms: Findings from a multi-site pilot study in Australia's Hunter Valley | Frontiers in Veterinary Science | 2022 | Hendra | Australia | Non-randomised controlled | 17 | NA | 71 | NR | Animal worker | 9 |
| Umapathi, T. et al | Progressive upper limb weakness - A delayed effect of Nipah virus infection? | Journal of the Neurological Sciences | 2009 | Nipah | Singapore | Case series/reports | 1 | 1 | 0 | 32 | Animal worker | 1 |
| Watanabe, S. et al | Effective inactivation of Nipah virus in serum samples for safe processing in low-containment laboratories | Virol J | 2020 | Nipah | Japan | Case-control | NR | NA | NR | NR | General pop. | 2 |
| Wiethoelter, A. K. et al | "We've learned to live with it"-A qualitative study of Australian horse owners' attitudes, perceptions and practices in response to Hendra virus | Preventive Veterinary Medicine | 2017 | Hendra | Australia | Cross-sectional | 27 | NA | 59 | 24-64* | Animal worker,Other (horse owners) | 9 |
| Wong, K. T. et al | Human Hendra virus infection causes acute and relapsing encephalitis | Neuropathol Appl Neurobiol | 2009 | Hendra | Australia | Case series/reports | 4 | 4 | 25 | Range: 35-49 | General pop. | 1 |
| Wong, S. C. et al | Late presentation of Nipah virus encephalitis and kinetics of the humoral immune response | Journal of Neurology Neurosurgery and Psychiatry | 2001 | Nipah | Indonesia | Case series/reports | 1 | 1 | 100 | 12 | General pop. | 5 |
| World Health Organisation | Nipah virus outbreak(s) in Bangladesh, January-April 2004 | Wkly Epidemiol Rec | 2004 | Nipah | Bangladesh | Case series/reports | 12 | 12 | 25 | NR | General pop. | 1, 4 |
| Yadav, P. D. et al | Nipah Virus Outbreak in Kerala State, India Amidst of COVID-19 Pandemic | Front Public Health | 2022 | Nipah | India | Case series/reports | 65 | 1 | 48 | Range: case 12; contacts 16-71 | General pop.,HCW | 1, 2, 4 |
| Yadav, P. D. et al | Standardization & validation of Truenat™ point-of-care test for rapid diagnosis of Nipah | Indian J Med Res | 2021 | Nipah | India | Case-control | 188 | 8 | NR | NR | General pop. | 4 |
| Yong, M. Y. et al | Seroprevalence of nipah virus infection in peninsular Malaysia | Journal of Infectious Diseases | 2021 | Nipah | Malaysia | Cross-sectional | 177 | 19 | 54 | Range: 3-75 | General pop. | 8 |
| Zhang, X. A. et al | A Zoonotic Henipavirus in Febrile Patients in China | N Engl J Med | 2022 | Langya | China | Case series/reports | 35 | 26 | 61 | Range: 9-76 | General pop. | 1, 2 |

- *Reported in age categories, so no clear age range or median/mean presented
- **Abbreviations: gen pop.=general population, HCW=healthcare workers, NR=not reported, NA=not applicable
- ***Domain 1: Clinical characteristics, 2: Transmission & prevention, 3: Vaccines, 4: Diagnostics, 5: Immune protection, 6: Therapeutics, 7: Supportive care, 8: Risk factors, 9: Social sciences.
